# Inebriate Asylums

**Published:** 1874-09

**Authors:** 


### INEBRIATE ASYLUMS.

These are institutions which aim to help those who are occasionally or habitually drunk to correct their habits and reform. Dr. Joseph Parrish, Secretary of the Maryland Inebriate Asylum, at Baltimore, in his last annual report to the Governor of Maryland, makes some suggestive statements of great practical value, in which every intelligent reader has a greater or less personal interest.

One-fourth of all the inmates had either reduced themselves to such poverty as to become pensioners on the bounty and benevolence of others, or had lost so much of their integrity and manliness as to leave the institution without paying anything, after having expressed their determination to do so on making application for admission. Item: Drunkenness either robs a man of his property and makes him a beggar, or destroys his high sense of honor, or makes him a remorseless liar or a mean swindler in a great many cases. The high-minded young man who, to-day, would scorn to do or even think a mean thing, runs a great risk of getting into the habit of doing both, if he only occasionally takes something to drink, if he only occasionally drinks a glass of wine.

For the encouragement of those who voluntarily place themselves within the rules of these institutions, it is stated that almost all derive some benefit if they remain even two or three months, while one-fourth were able afterwards to embark in successful business. Some are dismissed as "incorrigible," so completely abandoned to vicious practices, that it is useless to make any further effort to lift them out of their degradation, and return, like "the sow that was washed, to her wallowing in the mire."

Months and even years are required to confirm habits of temperance, before it is safe to place them within sight or sound, or reach of the "accursed thing." This report goes on to say that very few are cured; that no reliance can be placed on the word of honor; that the perverted ingenuity of the insane is exercised in evading the watchers and procuring drink in an underhand manner, and that they cannot be trusted with a dime to go into the town, yet these things are so in a great many cases; and further, no man is safe from an ignominious fall, even after years of abstemiousness, who has ever been so unfortunate as to drink excessively. One encouraging remark worthy the special attention of those who are so unfortunate as to have fallen into intemperate habits, that if persons are now and then found who, by their own will and determination, have reformed permanently, there is reason to believe that in any given case the chances of reformation are increased, when in addition to their own wishes and efforts to reform, all the aids of a well managed institution are superadded.

A loud warning is given in this admirably successful and comprehensive report to that large class of young men found in all large cities called

CLERKS.

There were three times as many clerks in the Maryland Institution last year as any other class. This is a fact which should arrest the attention of all our Young Men's Christian Associations, and should be often prominently brought forward. The germs of these habits are formed in New York City on the occasion of New Year's calls and the baleful habit of taking one meal a day at eating houses, many of which advertise "Free Lunch," but it is the understanding that it would be considered "mean" not to call for something to drink, the profits on which, on an average, are greater than the cost of what was eaten. A most laudable effort has been made to break up this baleful practice, in the establishment of

QUAKER DAIRIES.

In several prominent places in New York City, there are furnished the purest, richest milk, and the lightest, whitest bread, and the tenderest, best cooked meats, and the most carefully baked and well seasoned pies, at reasonable rates, and everything faultlessly clean and tidy, and respectable and home-like. The New York *Daily Witness* deserves the thanks of this whole community for being the first newspaper to bring these Quaker Dairies into public notice and to commend them to the patronage of all respectable merchants and merchants' clerks. Let all city parents see to it that their sons, if they must lunch down down, patronize these places, and let it be to all young ladies a test for the steadiness and real worth of a young man, that he lunches at a Quaker Dairy.

